# Social parasitism and the molecular basis of phenotypic evolution

**DOI:** 10.3389/fgene.2015.00032

**Published:** 2015-02-18

**Authors:** Alessandro Cini, Solenn Patalano, Anne Segonds-Pichon, George B. J. Busby, Rita Cervo, Seirian Sumner

**Affiliations:** ^1^Dipartimento di Biologia, Università di FirenzeFirenze, Italy; ^2^Institute of Zoology, Zoological Society of LondonLondon, UK; ^3^The Babraham Institute, Babraham Research Campus – CambridgeCambridge, UK; ^4^Wellcome Trust Centre for Human GeneticsOxford, UK; ^5^School of Biological Sciences, University of BristolBristol, UK

**Keywords:** phenotypic plasticity, social insects, *Polistes*, social parasites, genomics, gene expression

## Abstract

Contrasting phenotypes arise from similar genomes through a combination of losses, gains, co-option and modifications of inherited genomic material. Understanding the molecular basis of this phenotypic diversity is a fundamental challenge in modern evolutionary biology. Comparisons of the genes and their expression patterns underlying traits in closely related species offer an unrivaled opportunity to evaluate the extent to which genomic material is reorganized to produce novel traits. Advances in molecular methods now allow us to dissect the molecular machinery underlying phenotypic diversity in almost any organism, from single-celled entities to the most complex vertebrates. Here we discuss how comparisons of social parasites and their free-living hosts may provide unique insights into the molecular basis of phenotypic evolution. Social parasites evolve from a eusocial ancestor and are specialized to exploit the socially acquired resources of their closely-related eusocial host. Molecular comparisons of such species pairs can reveal how genomic material is re-organized in the loss of ancestral traits (i.e., of free-living traits in the parasites) and the gain of new ones (i.e., specialist traits required for a parasitic lifestyle). We define hypotheses on the molecular basis of phenotypes in the evolution of social parasitism and discuss their wider application in our understanding of the molecular basis of phenotypic diversity within the theoretical framework of phenotypic plasticity and shifting reaction norms. Currently there are no data available to test these hypotheses, and so we also provide some proof of concept data using the paper wasp social parasite/host system *(Polistes sulcifer—Polistes dominula)*. This conceptual framework and first empirical data provide a spring-board for directing future genomic analyses on exploiting social parasites as a route to understanding the evolution of phenotypic specialization.

## Introduction

### The molecular basis of phenotypic diversity

Evolution plays with inherited traits to produce altered phenotypes which may be better adapted to fill a niche different to that of their ancestors. Ultimately, phenotypic traits arise at the level of the genes. A major outstanding question in evolutionary biology is what roles do losses, gains, co-options and modifications of genomic material play in the evolution of phenotypic diversity within and between species? (West-Eberhard, 2003; Kaessmann, 2010; Van Dyken and Wade, 2010; Wissler et al., 2013) Many species show phenotypic plasticity in the expression of alternative phenotypes from the same genotype, through variance in reaction norm responses to changes in the environment (Aubin-Horth and Renn, 2009). Such plasticity affects both short-term (ecological) and long-term (evolutionary) adaptation, and thus influences survival and fitness (Pfennig et al., 2010; Beldade et al., 2011; Hughes, 2012). Conditional expression of the genes underlying polyphenisms facilitate gene, and consequently phenotypic, evolution (Van Dyken and Wade, 2010). Canalized developmental pathways shaped by evolution can result in heritable shifts in phenotype (Waddington, 1942). Genomic methods in modern evolutionary biology now allow us to dissect the molecular basis of such phenotypic diversity across a range of organisms, from genes to phenotypes (Tautz et al., 2010). But selection acts directly on phenotypes and only indirectly on the molecular machinery, and so an integrated study of key phenotypic traits in ecologically relevant settings and the genes associated with them is essential (West-Eberhard, 2005; Schwander and Leimar, 2011; Valcu and Kempenaers, 2014). Insects provide excellent models for studying these facets of phenotypic evolution within and across species (Nijhout, 2003; Moczek, 2010; Simpson et al., 2011), e.g., eusocial insect castes (Evans and Wheeler, 2001; Smith et al., 2008), male morphologies beetles (Moczek, 2009), asexual and sexual reproductive phases in aphids (Brisson and Stern, 2006).

An ideal model system for determining the molecular basis of phenotypic evolution allows comparisons of related species which have evolved mutually exclusive traits and/or life histories (e.g., Arendt and Reznick, 2008; Schlichting and Wund, 2014). Parasites are good examples of species that have lost ancestral, free-living traits and gained new ones to evolve a specialized life-history that depends on exploiting the resources of other species. For example, endoparastic worms have lost ancestral gut, head and light sensing organs, but have gained traits such as a specialized tegument, which protects them from host-stomach acids (Burton et al., 2012). Hosts co-evolve to combat parasitism, through enhanced immune responses and mechanisms for detecting infection; parasites manipulate their host to benefit the parasite's life cycle, often through an extended phenotype (Dawkins, 1982). Comparisons between parasites and their free-living relatives therefore present intriguing models for studying the molecular basis of phenotypic evolution (Dybdahl et al., 2014). However, these comparisons are complicated by co-evolution where frequency distributions of host and parasite genotypes (and traits) shift reciprocally and responsively over time, and moreover hosts and their parasites are rarely closely related species (Hamilton, 1980).

Insect social parasites and avian brood parasites differ from other parasites in that they exploit the parental behavior of the hosts rather than the physical resources of individuals. Such parasites have evolved several times in the animal kingdom. For example, cuckoldry occurs in more than 100 bird species, where the host pays the cost of raising unrelated chicks (Davies, 2000). Social parasites of eusocial insects (e.g., the Hymenoptera—bees, wasps and ants) are especially interesting as they are usually close relatives of their hosts, and have often entirely lost their worker caste (Savolainen and Vepsäläinen, 2003). The potential for using social parasites, especially of eusocial insects, as models for understanding the molecular basis of phenotypic plasticity has been recognized (West-Eberhard, 1989, 2003). However, we lack a defined theoretical framework and clear hypotheses to properly exploit this untapped niche using molecular studies. Advances in molecular technologies now make gene-level studies accessible in any organism. It is therefore timely to lay out a framework for exploiting social parasites and their hosts as models for understanding the genomic basis of phenotypic losses and gains in evolution. Here we identify the key traits of hymenopteran social parasites of eusocial insects that make them useful models for understanding phenotypic evolution at the molecular level. We define specific, testable hypotheses on the molecular basis of shared and contrasting traits in the evolution of social parasitism within the conceptual framework of shifting reaction norms and phenotypic plasticity (e.g., Aubin-Horth and Renn, 2009; Fusco and Minelli, 2010). We also provide a first test of some of these hypotheses, as proof of concept for our conceptual model and a spring-board for future genomic analyses on the evolution of phenotypic adaptation (see Supplementary Materials).

### Social parasitism in eusocial insects

There are over 14,000 eusocial species in the Hymenoptera (bees, wasps and ants) representing over 11 independent origins of eusociality. Their societies are defined by a division of reproductive labor in the form of queen and worker castes, overlapping of generations, and cooperative brood care. Social parasitism has evolved multiple times independently in the eusocial insects: three times in wasps (once in Polistinae *Polistes*—Choudhary et al., 1994; Cervo, 2006; twice in Vespinae—genus *Vespula* and *Dolichovespula*, Carpenter and Perera, 2006); at least 12 times in bees [three times in bumblebees - *Bombus* (subgenus *Psythrus*, Thoracobombus) and *Alpinobombus*, (Alford, 1975; Cameron et al., 2007; Hines and Cameron, 2010)]; seven times in Allodapinae (Tierney et al., 2008; Smith et al., 2013); twice in Halictidae (*Dialictus* genus, Gibbs et al., 2012); and multiple times in the ants (Huang and Dornhaus, 2008; Buschinger, 2009).

There are several features of social parasite/host systems that make them ideal models for studying the molecular basis of phenotypic diversity. Their easily observable behaviors (e.g., paper wasps *Polistes*, Cervo, 2006, and leafcutting ants *Acromyrmex;* Sumner et al., 2004a) facilitate an integrated study of the behavioral phenotype with the molecular one. Social parasites are usually closely related to their hosts and thus share recent genomic (and phenotypic) ancestry (Box 1) (Choudhary et al., 1994; Lowe et al., 2002; Savolainen and Vepsäläinen, 2003; Sumner et al., 2004a; Huang and Dornhaus, 2008; Smith et al., 2013). Obligate social parasites depend on their host for their entire life cycle, and so have lost many of the essential free-living traits such as the ability to found a nest, produce an effective worker caste and raise offspring (Sumner et al., 2004b; Cervo, 2006; Buschinger, 2009). They have also evolved new traits, e.g., the ability to manipulate the host worker force so that parasitic offspring are raised as if they were host offspring. Full release from free-living traits means there are few restrictions on phenotypic evolution. This may facilitate phenotypic diversity at the molecular level. Obligate social parasites of eusocial insects therefore allow a direct comparison of the molecular basis of traits with recent, shared evolutionary history and contrasting traits that have evolved (and persist) within the same environmental context (see Box 1).

Box 1 **Obligate social parasites and their hosts as models.**

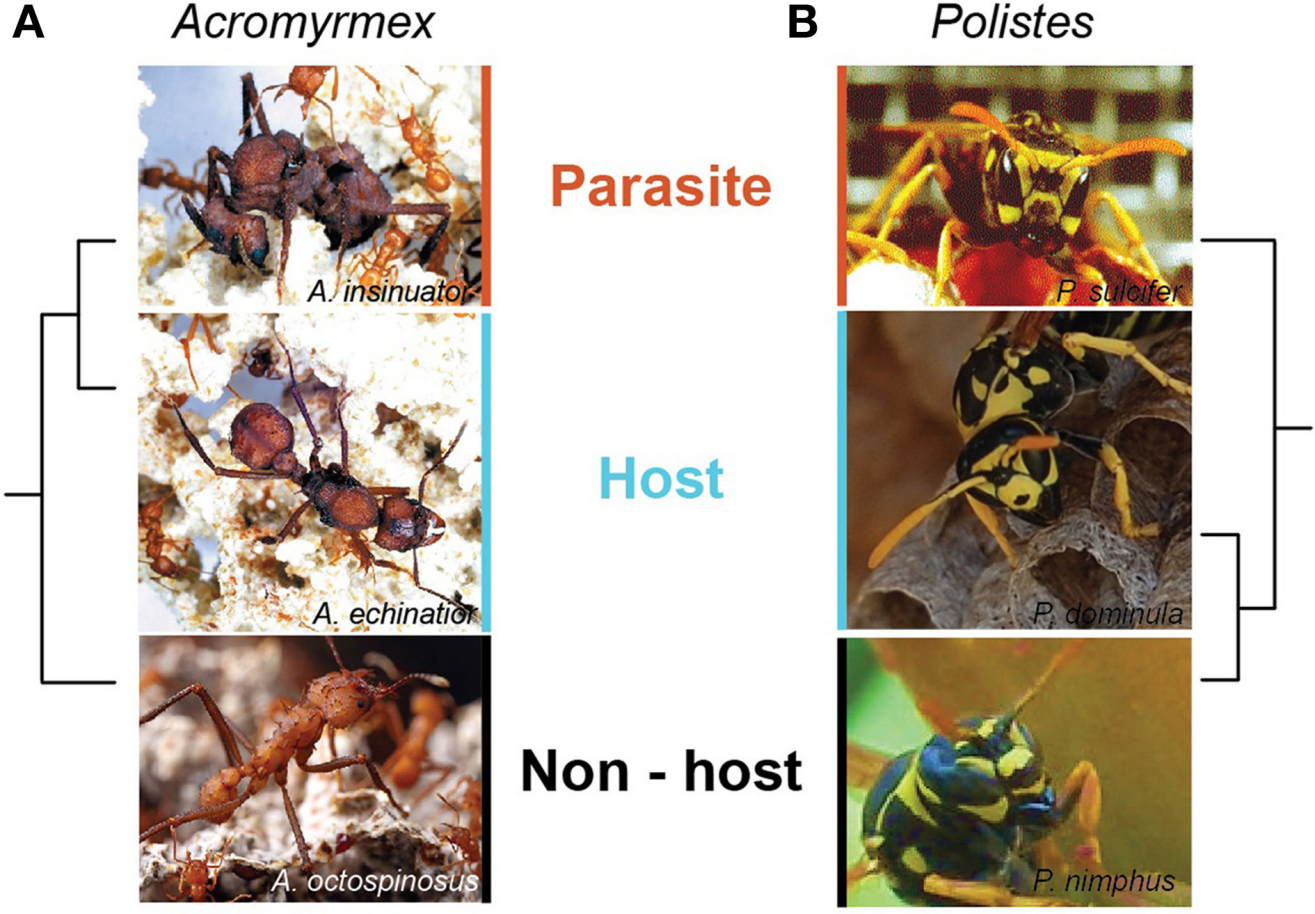

There are several features unique to hymenopteran obligate social parasite/host systems that make them ideal models for studying the molecular basis of phenotypic diversity.

1. Close phylogenetic relationships. Social parasites of hymenopterans are usually close relatives of their host, in a strict **(A)** or loose **(B)** sense, and thus share recent genomic ancestry (Lowe et al., 2002; Savolainen and Vepsäläinen, 2003; Sumner et al., 2004a; Huang and Dornhaus, 2008; Smith et al., 2013). Hosts are likely to represent the eusocial ancestor of the parasite at the molecular and phenotypic levels, providing the opportunity to compare how the parasite has diverged from its ancestral state.
2. Non-host sister species. Intriguingly, sister species of hosts are often resistant to their relative's social parasite, despite occurring sympatrically and sharing similar ecologies, phenotypes, life histories and environments. It is not known how non-hosts confer resistance, but comparisons of host and non-hosts at the molecular level may shed light on this.
3. Cryptic morphology. Although hymenopteran social parasites differ significantly to their hosts in life strategy and behavior, they are usually near indistinguishable from their hosts morphologically e.g., (**A)**: *Acromymex insinuator* (social parasite), *Acromyrmex echinator* (the host), and *Acromyrmex octospinosus* (non-host sister species); **(B)**: *Polistes sulcifer* (social parasite), *Polistes dominula* (the host) and *Polistes nimphus* (non-host sister species). This is important for genomic analyses of phenotypic plasticity where we are interested in understanding the molecular basis of traits other than morphology (e.g., behavior). Shared morphology between parasite and host therefore helps to controls to some extent for the machinery underlying morphological differences. Molecular analyses also help with social parasite species discovery, as the parasites may be cryptic at the morphological level, but not at the molecular level.
4. Trait losses and gains. Because both social parasite and host can be easily observed within and out of the nest, phenotypic traits can be easily identified, quantified and compared. Social parasites lack a wealth of free-living traits (e.g., maternal care, provisioning, nest-founding), but also exhibit novel traits (e.g., fighting ability, usurpation behaviors, chemical mimicry, cryptic manipulation). Whilst these are well studied at the phenotypic level, we know nothing about how such losses and gains occur at the molecular level. Comparisons of the molecular bases of closely related host and social parasite traits will provide new insights into phenotypic evolution.

Photo credits: Alessandro Cini, Rita Cervo, Stefano Turillazzi and David Nash.

## A model

**Figure 1:**
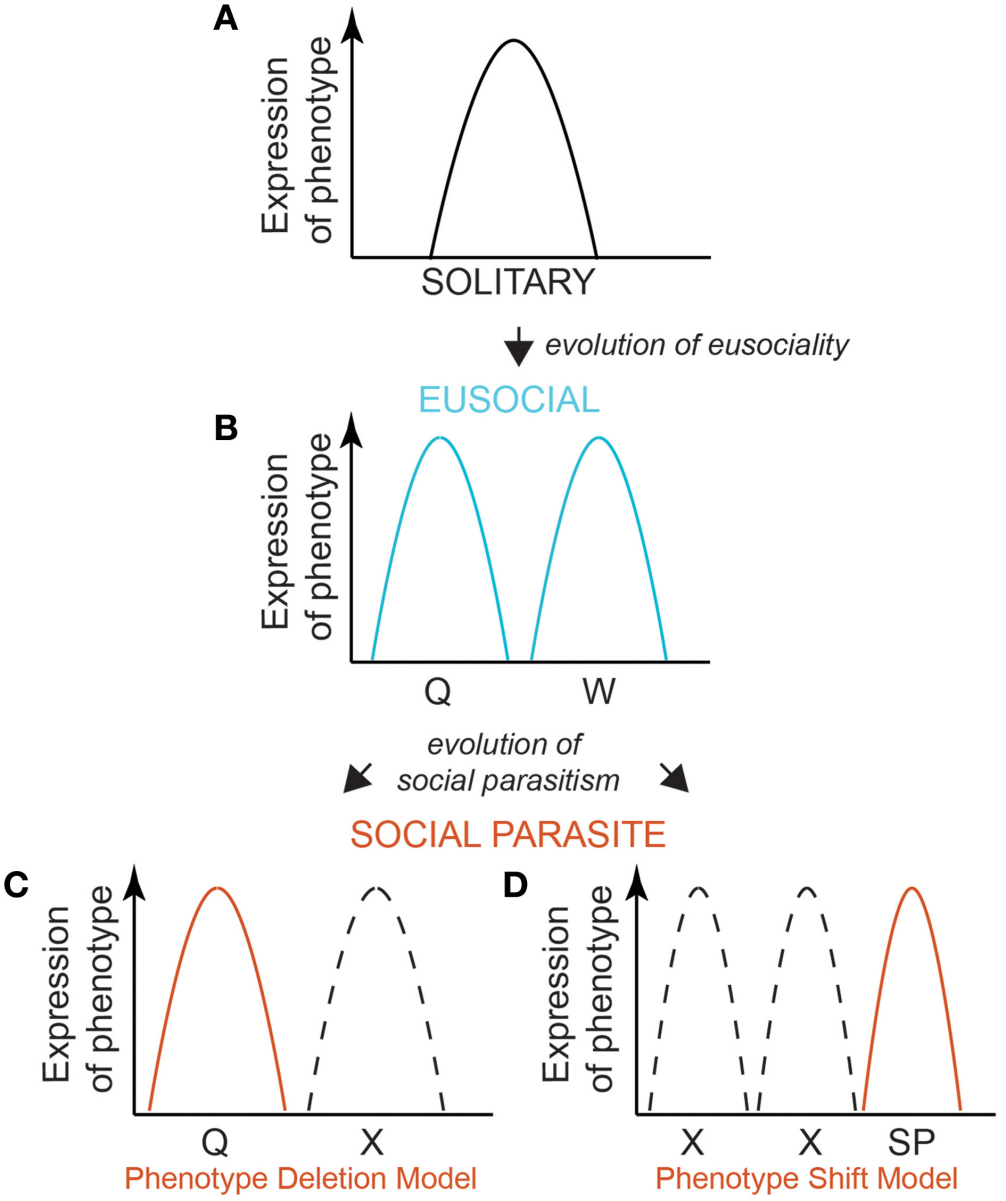
**A model for the evolution of a social parasite phenotype from a eusocial ancestor**. A model of shared and contrasting reaction norms is a useful way of exploring the possible ways by which social parasite phenotypes may evolve. A bell curve describes the expression of a single phenotype in a solitary species **(A)**. Eusocial insects evolved form a solitary ancestor **(A)**, and produce two phenotypes—reproductive queens and non-reproductive workers **(B)**. Queens and workers occupy a bimodal expression of phenotypic space, expressing distinct mutually exclusive molecular phenotypes (e.g., gene expression profiles). The genome remains plastic and able to produce alternative phenotypes in response to the environment. Social parasites may evolve via canalization, whereby the phenotype is fixed (as a reproductive) irrespective of the environment, and so unlike its eusocial ancestor, phenotypic expression is robust to the environment: social parasites always produce a reproductive and never a worker phenotype. We propose two ways by which this could arise. Since the social parasite resembles so closely the phenotype of their ancestral eusocial queen, one model is that the worker phenotype is functionally “deleted.” This would suggest that the phenotypic reaction norm landscape of the worker caste is not expressed (**C**, Phenotype Deletion Model). An alternative is that the social parasite is a new, or modified, phenotype, with a reaction norm that is different to both the bimodal (caste) peaks of the eusocial ancestor (**D**, Phenotype Shift Model). For simplicity, we place this shifted phenotype in a different phenotypic space to the ancestral queen and worker phenotypes, but this curve could lie at any point. Dashed curves depict the ancestral eusocial phenotypes that are no longer expressed by the social parasite. Determining this point may shed light on the mechanisms of social parasite phenotype evolution. The two models are not necessarily mutually exclusive: depending on the time since divergence between the lineages, the two models may represent different ends of a spectrum of phenotypic evolution.

Eusocial species evolve from solitary ancestors. Solitary phenotypes occupy a normal distribution of variation, determined by their individual threshold level of response to environmental cues (Figure 1A). Queen and worker castes are alternative phenotypes that arise from the same genome, via bi-modal developmental pathways of individuals with evolved differences in their response thresholds to an environmental cue (Wheeler, 1986; Nijhout, 2003; Page and Amdam, 2007; Figure 1B). These alternative phenotypes arise through differential expression of shared genes, possibly via epigenetic regulation (Sumner, 2006; Smith et al., 2008; Patalano et al., 2012; Yan et al., 2014). This bi-modal landscape of phenotypic fitness is the ancestral basis from which social parasites must evolve. There are two likely routes by which specialized social parasites evolve from their eusocial ancestor. They may lose the worker phenotype and thus share a phenotypic fitness landscape with just the queens of their social ancestor (De Visser and Krug, 2014). Their phenotype response therefore becomes genetically fixed (canalized) by genetic assimilation, with selection favoring the loss of plasticity such that the genotype no longer responds to the caste-relevant environmental cue (“Phenotype Deletion Model” Figure 1C). Alternatively, they may evolve an entirely new phenotype with a novel/contrasting phenotype-response curve (“Phenotype Shift Model” Figure 1D), by genetic accommodation whereby there is selection for altered patterns of gene expression and associated phenotypic effects (West-Eberhard, 2003; Schlichting and Wund, 2014). Under either scenario, the pre-existing polyphenism of the eusocial ancestor facilitates the evolution of the specialist social parasite. Determining which route evolution takes is important: in the Deletion Model (Figure 1C) co-option of conserved genomic processes would be paramount, but with silencing of the worker response threshold (e.g., using the existing machinery used by queens to silence worker expression); in the Phenotype Shift Model (Figure 1D) novel genomic processes (e.g., brought about via mutation) would be important in generating a new range of response thresholds to the environment. The timing since speciation between the eusocial ancestor and social parasite is also important to consider as this may mean the two models are not mutually exclusive: the longer the time since the two lineages split, the more differences each lineage may accumulate. There may be a transition from the phenotype shift model to the phenotype deletion model for traits, gene expression or genes, depending on the time since the two lineages split.

## Hypotheses and predictions

**Figure 2:**
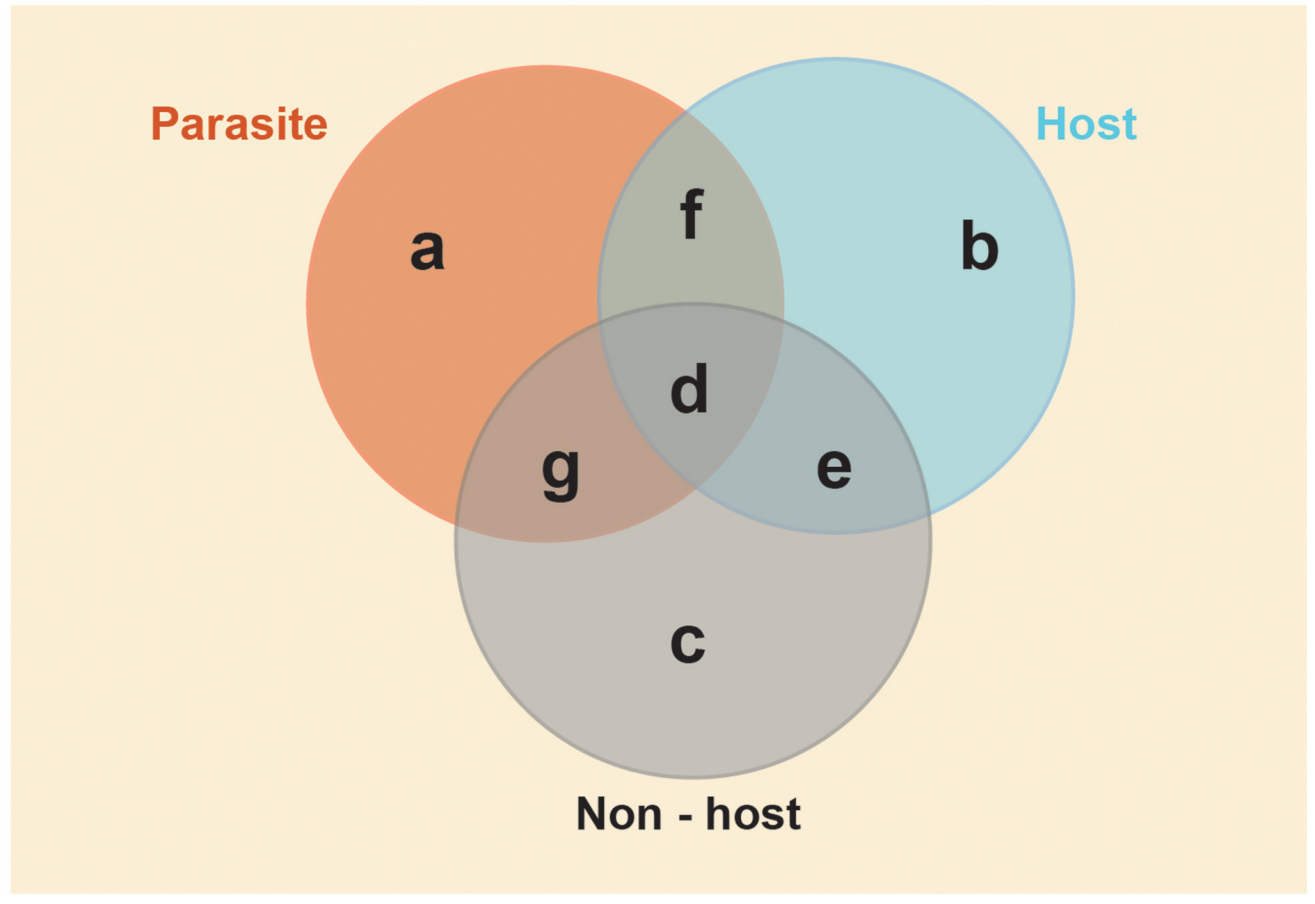
**Conceptual framework for predictions on shared and contrasting genomic/phenotypic diversity in social parasite/host relationships**. Venn diagram depicting predicted shared and contrasting molecular phenotypes of non-hosts, hosts and social parasites. We define the molecular phenotype to include contrasting patterns of gene expression (significant up or down regulation), gene regulatory elements (e.g., non-coding RNAs, microRNAs, DNA methylation, histone modifications), gene interaction networks (e.g., correlated co-expression) and protein synthesis. Each area represents the molecular phenotype of the specific suite of traits. Overlapping areas indicate putatively shared molecular phenotypes. The yellow shaded area indicates the shared environment of the three species, which we predict will cause similar responses in molecular phenotypes of all three species. Conserved generic traits (area d): Molecular processes underlying traits shared by all species, and thus putatively inherited from their common ancestor. These will include fundamental machinery for growth, cell repair, metabolism, as well as more specific traits of interest that are shared among queens and social parasites such as aggression and reproduction. Identifying the molecular phenotype of this area allows tests of the genetic toolkit hypothesis. Parasite-specific (area a): Molecular processes underlying traits that have evolved in the parasite to facilitate its specialized parasitic life style, for example enhanced fighting ability, usurpation behaviors, cryptic mimicry. Identifying the molecular phenotype of this area addresses the question of whether newly evolved phenotypic traits require new genes/pathways or simply re-use existing ancestral genes/pathways. Free-living specific (area e): Molecular processes underlying free-living traits that no longer provide a fitness advantage to social parasites, e.g., parental care traits and nest founding. Identifying the molecular phenotype of this area allows us to determine what happens at the molecular level when phenotypic traits are lost, e.g., are there changes in regulation/expression, loss of processes/genes? Restricting this to traits/genes shared by free-living host and non-host species is likely to represent the traits present in the eusocial ancestor of the social parasite, and exclude processes that may have evolved subsequently. These latter processes may be associated with social parasite resistance (areas c and g) in sympatric non-hosts, host response to parasitism (area b) and co-evolved traits (area f) in host and parasite that are absent from the non-host.

Here we present some testable hypotheses for these models. These hypotheses and predictions are specific to obligate social parasites and their eusocial insect hosts, but they may also be of general relevance to furthering our understanding of the molecular basis of phenotypic diversity. The empirical approach we suggest requires a combined analysis of individual-level behavioral monitoring with subsequent quantitative analyses of the many components of the molecular phenotype (Pavey et al., 2010), e.g., transcription (RNAseq/transcriptomics; Ferreira et al., 2013), protein synthesis (proteomics; Begna et al., 2012), regulatory elements (e.g., microRNAs; Greenberg et al., 2012) and epigenetic modifications (Kucharski et al., 2008; Lyko et al., 2010; Bonasio et al., 2012; Simola et al., 2013). In Figure 2, we illustrate schematic regions of shared and contrasting trait-associated molecular phenotypes, which we refer to in our hypotheses, and suggest this as a useful way of making sense of complex genomics datasets.

### Hypothesis 1: conserved molecular processes underlie convergent phenotypes

Conserved genes, like the *Hox* gene family (Lee et al., 2003; Fernald, 2004), underlie convergent phenotypes, suggesting that phenotypic variation can evolve using shared genes and regulatory mechanisms differently (Shubin et al., 2009; Stern, 2013). By this mechanism, evolution re-uses the same ingredients (or “toolkit”) in different organisms, but tinkers with the recipe to produce different outcomes. By expressing genes at different times in development and/or in different parts of the body, the same genes can be used in different combinations, generating phenotypic diversity and innovation. Animals look different not because the molecular machinery is different, but because different parts of the machinery are activated to differing degrees, at different times, in different places and in different combinations. The number of combinations is huge, and so this is a compelling and simple explanation for the development of complex and diverse phenotypes from even a small number of genes. For example, the human genome has a mere 19,000 protein-coding genes (Ezkurdia et al., 2014), and yet humans are arguably one of the most complex products of evolution, and differ in significant ways from close relatives with similar gene sets. “Toolkit” genes are old, present in all animals and often share functions across species. Conserved toolkit genes associated with convergent social behaviors have been detected in a range of eusocial insects (Toth and Robinson, 2007; Fischman et al., 2011; Woodard et al., 2011; Toth et al., 2014), but recent work has also revealed that eusocial lineages also harbor novel (taxonomically restricted) genes that are associated with eusocial behaviors (Ferreira et al., 2013; Simola et al., 2013; Feldmeyer et al., 2014; Sumner, 2014).

Closely related social parasites and their hosts are especially powerful models for asking to what extent conserved molecular processes underlie similar phenotypes in species with shared, recent genomic inheritance. The toolkit hypothesis predicts that host queens and social parasites will share the same molecular phenotype (i.e., express the same genes and proteins), because they are both reproductive specialists. Support for this hypothesis would suggest that social parasites are simply a reduced form of the social phenotype, expressing the reproductive component, but suppressing the worker component of their ancestors (i.e., the Phenotype Deletion Model; Figure 1C). Alternatively, if gene conservation is not supported, this may suggest that social parasitism evolves via Phenotype Shift (Figure 1D), or a combination of the two processes. This can be tested by looking at shared transcriptional patterns between social parasites and their host queens (See Figure 2; molecular processes underlying traits in areas d & f).

**Figure 3:**
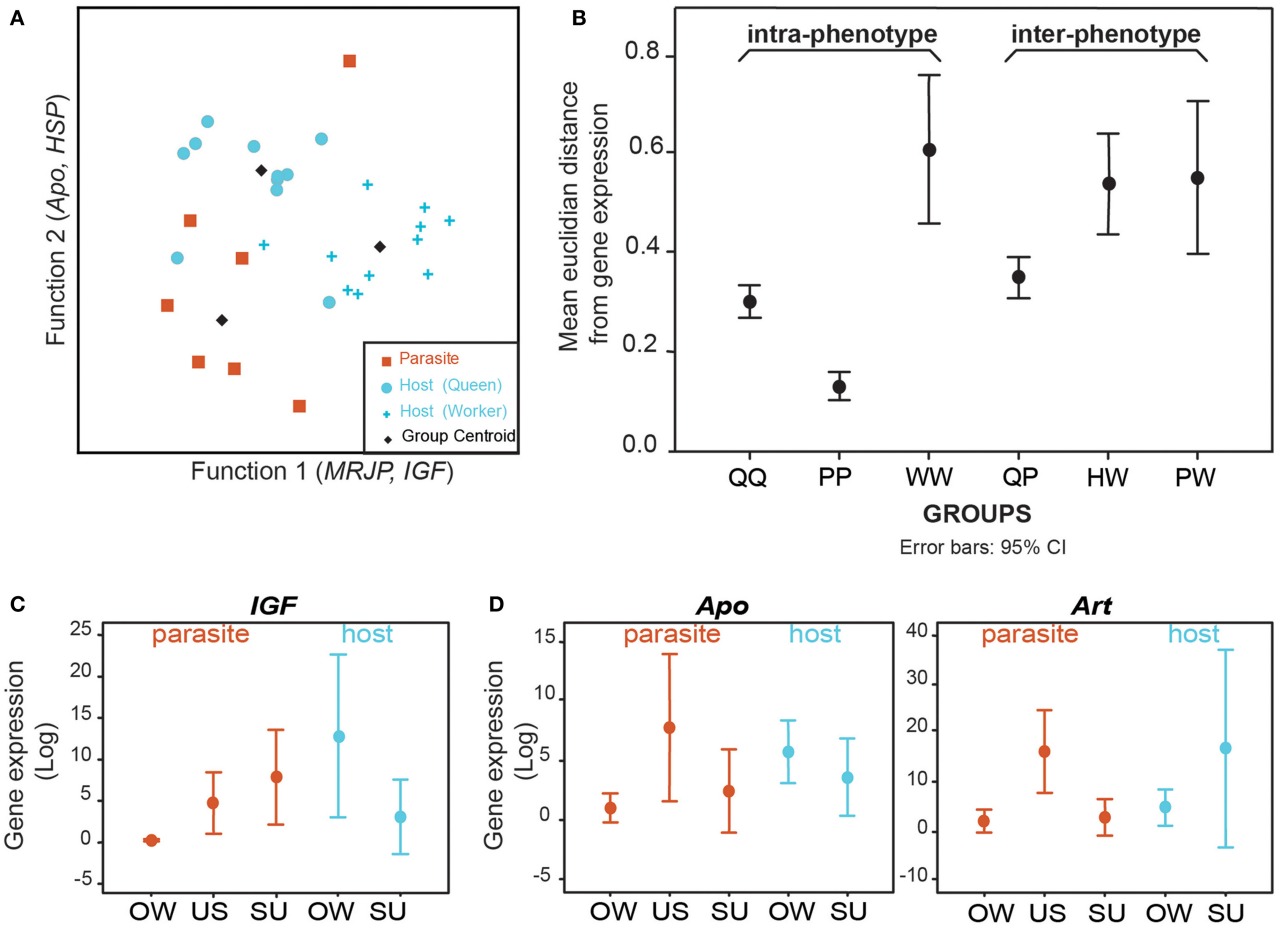
**Brain gene expression data from the social parasite *Polistes sulcifer* and its social host *Polistes dominula***. Comparison of expression levels for five “toolkit” genes that are differentially expressed among queens and workers in *Polistes* (chosen from: Sumner et al., 2006; Toth et al., 2007; Ferreira et al., 2013). *Arrestin* (*Art*) is expressed in response to light; *Apolipophorin* (*Apo*) is involved in general metabolic processes and lipid transport; *Heat Shock Protein 70kDa* (*HSP*) is involved in response to heat stimulus; insulin growth factor (IGF) responds to nutrition; *Major Royal Jelly Protein* (*MRJP*) is a yellow protein associated with reproductive behaviors. We compared individual-level gene expression across three phenotypes: social parasites (P), host queens (Q) and host workers (W). **(A)** Discriminant analyses revealed three distinct clusters, corresponding to the 3 phenotypes. Function 1 closely correlates with gene expression of *MRJP* and *IGF*, and discriminates between social parasites and workers while function 2 closely correlates with *Apo* and *HSP* and discriminates social parasites from queens. 79.3% of individuals grouped into non-overlapping clusters. Cross validation analyses correctly classified 69% of samples. **(B)** Euclidean distances in gene expression among phenotypes showing greater inter-phenotype differences than intra-phenotypes (*t*-test, *t* = −2.114, *df* = 376, *p* = 0.035, *n* = 126 vs. 252). Gene expression differences between social parasites and queens were greater than among social parasites (Mann Whitney test, *U* = 233, *p* = 0.0005, *n* = 72 vs. 15). **(C,D)** Gene expression dynamics across the seasons (OW, overwinter; US, usurpation; SU. summer). **(C)** Changes in social environment experienced by the social parasites are accompanied by changes in *IGF* gene expression (within social parasites: Mann Whitney test, *U* = 4.0, *p* = 0.0183, *n* = 8 vs. 5; between species: Mann Whitney test, *U* = 8.0, *p* = 0.1498, *n* = 7 vs. 5). **(D)** *Apo* and *Art* are upregulated during usurpation compared to the pre and post usurpation periods (Kruskal Wallis test, *Apo*: *H* = 8.525, *p* = 0.0141: *Art*: *H* = 8.842, *p* = 0.0120). Expression levels of *Apo* and *Art* are significantly higher in usurping social parasites than in overwintering social parasites but no differences occur between overwintering and summer period [*Apo*: Mann Whitney post hoc pair wise comparisons US vs. OW *p* = 0.0112, US vs. SU, *p* = 0.0230; OW vs. SU *p* = 0.341, *n* = 9 (OW) vs. 5 (US) vs. 7 (SU), *Art*: Mann Whitney post hoc pair wise comparisons US vs. OW, *p* = 0.00848, US vs. SU, *p* = 0.01421; OW vs. SU *p* = 0.9485, *n* = 8 (OW) vs. 4 (US) vs. 6 (SU)]. No changes were observed in the expression levels for *Art* and *Apo* in the host species (Mann Whitney test, *Apo*: OW vs. SU Hosts *U* = 12,0, *p* = 0.2343, *n* = 7 vs. 6; *Art*: *U* = 14.0, *p* = 0.366, *n* = 7 vs. 6). No significant changes in *MRJP* and *HSP* gene expression dynamic across season were observed in parasites (Mann Whitney test, *MRJP*: *U* = 4, *p* = 0.176; *HSP*: *U* = 13.0, *p* = 0.236), or in the hosts who remain in a social environment throughout (Mann Whitney test, *MRJP*: *U* = 8.0, *p* = 0.246; *HSP*:*U* = 6.0, *p* = 0.226) (data not shown).

Preliminary data suggest that expression of toolkit genes is not conserved in the evolution of a social parasite, supporting the Phenotype Shift Model (Figure 1D). Analyses of gene expression profiles for putative toolkit genes thought to be important in castes of *Polistes* paper wasps reveal that social parasites and their host queens have distinct expression patterns (Figure 3A, see Supplementary Materials). This is unlikely to be a species-level effect since host workers are equally as distinct from their conspecific queens (Figures 3A,B). Importantly, gene expression differences between social parasites and queens were greater than among social parasites, suggesting that social parasite gene expression is not strongly overlapping with the queens among these putative toolkit genes (Figure 3B). Quantitative transcriptome sequencing (e.g., RNAseq) would allow a comprehensive test of this. However, these preliminary data suggest that social parasites evolve via a Phenotype Shift Model (Figure 1D), and that they may be a more complex phenotype than simply a partial genomic expression of the ancestral social state (as suggested by the Phenotype Deletion Model, Figure 1C). We predict that the shared molecular components between host and parasite will be few and limited to fundamental processes, e.g., egg production and protein storage, as characteristics of any reproductively active insect.

### Hypothesis 2: conserved molecular processes underlie response to a shared environment

Molecular phenotypes (e.g., gene expression, regulation and protein synthesis) are highly labile and can change responsively to environmental variation. A key question is whether different organisms use the same genes to respond to the same environmental cues. There will be strong selection for the social parasites to be able to accurately detect and respond to their host's environmental cues since they share the same intimate environment on the nest. Moreover, the social parasite must synchronize its life cycle and behavior perfectly with the host's life cycle (Cervo, 2006; Ortolani et al., 2008). The molecular processes underlying responsiveness to their shared environment may therefore be conserved. The Phenotype Deletion Model (Figure 1C) makes the implicit assumption that the phenotypes of host and parasite arise via different responses to the *same* environmental cue. Conversely, the Phenotype Shift Model (Figure 1D) is compatible with either a response to the *same* cue (but with a novel threshold), or a response to a *new* cue (i.e., one that evokes no caste-related response in the eusocial host).

One important phenotype-environment response trait in both hosts and social parasites is the ability to respond to the switch from a solitary to social environment. Many eusocial insects have a solitary phase, when a single queen founds a new colony and raises her first brood alone, and then switches to a eusocial phase when her workers emerge (see Box 2). Likewise, social parasites have a solitary phase, during which they need to locate and successfully infiltrate a host colony, followed by a social phase where the parasite takes over the role of the queen in a society of host workers (see Box 2). The Phenotype Deletion Model predicts that the social parasite co-opts the molecular plasticity of its eusocial ancestor. Thus, we would expect the same genes to change in both the social parasite, its eusocial host and any co-occuring related eusocial non-hosts (see Box 1) when each shifts from a solitary to a eusocial phase. In Figure 2 the social environment is depicted by the yellow shaded area surrounding the three species spheres. Since all three species (social parasite, host and non-host) occupy similar societies, we predict that each will respond to a shift between solitary (nest founding/nest searching) and eusocial (established queens on host/non-hosts, and established parasite queens on host colonies) environments using similar changes in their molecular phenotypes. Conversely, if the social parasites evolve via Phenotype Shifting, we would not necessarily expect host and social parasite to respond to the same cue, using the same molecular processes. A test of this requires comparisons of transcription, protein synthesis and regulatory elements in the solitary and eusocial forms of the reproductive phenotypes in each species (Figure 2, area d).

Box 2 **An example test system: the paper wasp social parasite *Polistes sulcifer* and its free-living host, the eusocial *Polistes dominula*.**

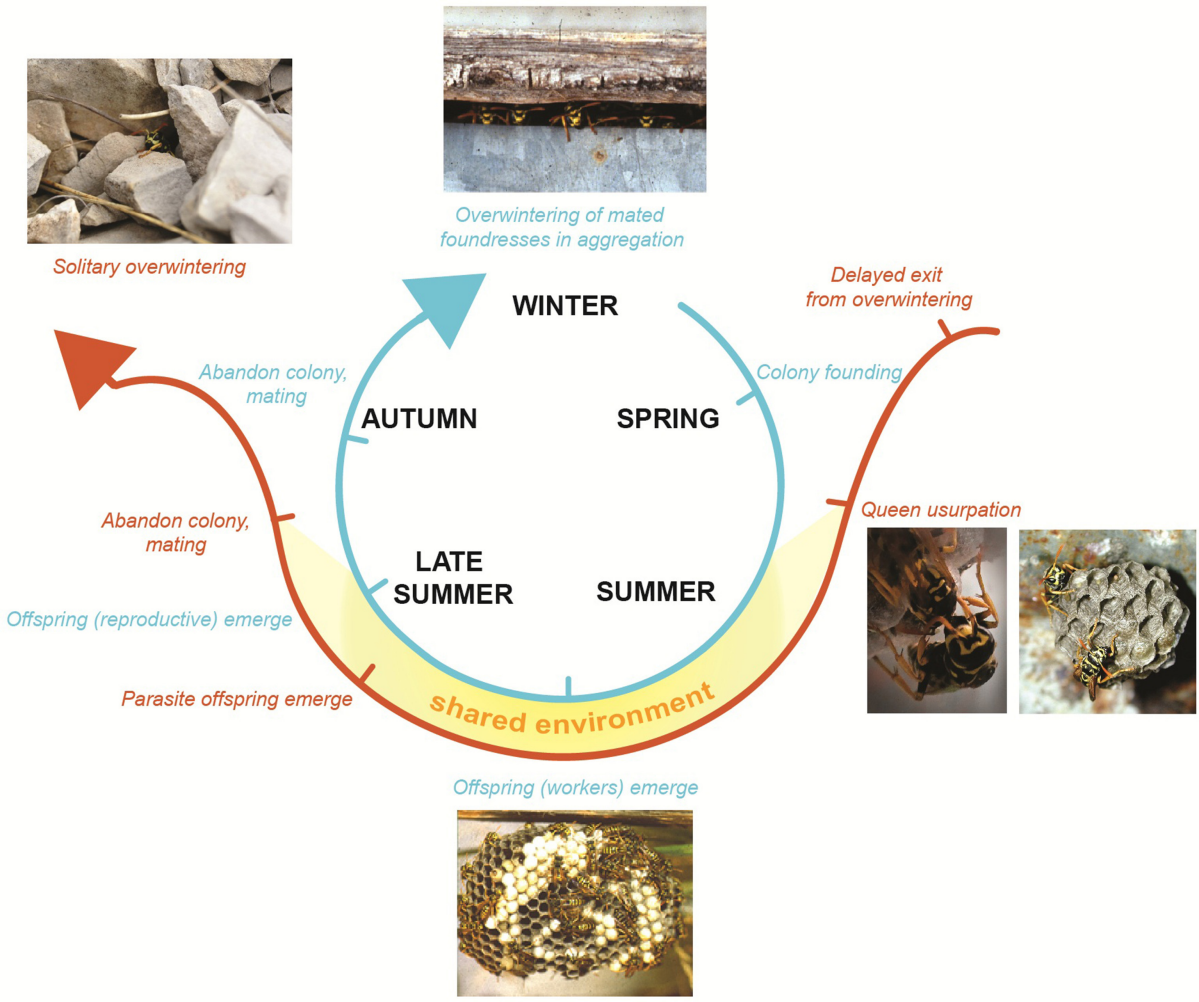

The molecular basis of phenotypes in Polistes has received some attention over the last few years (Sumner et al., 2006; Toth et al., 2007, 2009, 2010; Daugherty et al., 2011; Ferreira et al., 2013). *P. sulcifer* is the obligate social parasite of its close relative, the primitively eusocial wasp *P. dominula* (Choudhary et al., 1994). The life-history and behaviors of the social parasite-host system Polistes sulcifer-Polistes dominula, are well known (reviewed in Cervo, 2006), but we lack molecular analyses on the social parasites.

Both species have an annual lifecycle (Pardi, 1996; Cervo, 2006). Host colonies (blue line) are founded in spring (March–April) by one or more foundresses, among which a reproductive hierarchy is soon established through the mean of dominance interactions (Pardi, 1946). The first brood emerges around the end of May or early June and develops into workers. At the end of the summer, reproductives (males and females) emerge on the nest, leave the colony and mate. Males die soon after mating. Mated females cluster together in sheltered places to overwinter. Those who survive overwinter found new colonies the following spring (Pardi, 1996). Parasite females (orange line) emerge later than their hosts (late May) from overwintering (Cervo and Turillazzi, 1996) and migrate from their overwintering sites to pre-emergence host nests (Cervo and Dani, 1996; Cervo, 2006). Parasites find host colonies using visual and chemical stimuli (Cervo et al., 1996; Cini et al., 2011a). Nest usurpation takes place during a small window of time (late May-early June) (Cervo and Turillazzi, 1996; Ortolani et al., 2008) and it involves violent fights between hosts and parasites (Turillazzi et al., 1990; Cini et al., 2011b). Parasites display a novel behavior during this time (restlessness) (Ortolani et al., 2008). If the parasite is successful she becomes the sole egg-layer of the nest, adopting both the behaviors and chemical signatures of the host queen (Turillazzi et al., 2000; Sledge et al., 2001; Dapporto et al., 2004). After colony usurpation, the social parasite and un-parasitised host queens share the same environmental and social conditions (temperature, microclimate, diet etc.). Photo credits: Alessandro Cini, Rita Cervo and Stefano Turillazzi.

Among the toolkit genes we analyzed, insulin growth factor *(IGF)* is a putative candidate gene for response to changes in the social environment. We observed up-regulation of *IGF* in social parasites brains when they shift from solitary to social living, whilst *IGF* shows no change in expression in the constant eusocial environments of the host (Figure 3C). In our *Polistes* test system (see Box 2), both host and parasite over-winter as newly mated queens, but the parasite overwinters alone whilst the host overwinters in socially active aggregations (Dapporto and Palagi, 2006; Cini and Dapporto, 2009). If social context influences gene expression, hosts should show no significant change in the expression of genes responsive to social environment since they remain in a social phase during the winter and summer. Conversely, social parasites shift between solitary (overwintering) and social phases, and expression of genes responsive to social environment should reflect this dynamic, as seen with *IGF* in our system (Figure 3C). Recent work in a free-living species of *Polistes* has highlighted the importance of social environment in gene expression (Toth et al., 2014). Further analyses will reveal whether host/non-host species in the solitary founding phase also show similar patterns of response to environment as found in the social parasite (Figure 2, area d). Other likely candidate genes for this response include juvenile hormone-binding proteins and hexamerins, which are up-regulated in gregarious/social forms relative to solitary phases in the migratory locust (Kang et al., 2004).

### Hypothesis 3: trait losses and gains will be reflected at the molecular level

Phenotypically, social parasites exhibit a functional deletion of parental care traits (West-Eberhard, 2003). It is this observation that forms the basis of the Phenotype Deletion Model (Figure 1C). At the molecular level, selection for the genes/gene functions associated with parental care will be relaxed as their expression no longer has any fitness consequence. Such genes may be subject to rapid evolution, loss or other modifications (Hunt and Carrano, 2010; Hunt et al., 2011). This means genomic changes can be fixed rather than conditionally expressed (Van Dyken and Wade, 2010). Genes identified as important in parental care in host species therefore, are predicted to be lost (or not expressed) in social parasites. These traits can be easily recognized in the host (Figure 2, area b), thus providing a base-line of “absent” traits to compare with in the parasite (Figure 2, area a). Comparisons of molecular phenotypes of social parasites and their host (and non-host) workers are promising routes to defining the genes, regulatory processes and pathways involved in parental care in free-living species. Such analyses would provide a test of the Phenotype Deletion Model, and it also raises intriguing questions regarding the fate of the molecular processes involved in ancestral maternal care: does the parasite lose these genes/functions? In what sense are they “lost”; via their coding potential? What are the molecular processes that prevent these ancestral molecular processes from being expressed?

The evolution of social parasitism is accompanied by release from the evolutionary constraints experienced by a free-living species (Sumner et al., 2004b). This may allow the evolution of new/modified traits, not found in their free-living ancestor (West-Eberhard, 2003). For example, exaggerated morphological traits that enhance a social parasite's fitness e.g., enlarged Dufours glands in Vespine social parasites (Jeanne, 1977); enlarged mandibles (Cervo, 1994; Cervo and Dani, 1996); specific usurpation behaviors in Polistine social parasites (Ortolani et al., 2008); reduced scopae and mouthparts in Allodapinae social parasites (Michener, 1970; Smith et al., 2013); specialized piercing mandibles in slave making ants (Buschinger, 2009). Other traits include mechanisms of effective manipulation and deception of the host, such as chemical insignificance to elude host recognition and chemical mimicry to integrate into the host colony (Lenoir et al., 2001; Bagnères and Lorenzi, 2010; Bruschini et al., 2010) or suppression of host queens/workers reproduction (e.g., Cervo and Lorenzi, 1996; Vergara et al., 2003). A key question is whether these novel traits arise through co-opted conserved molecular processes, or via *de novo* birth of novel genes and/or re-organization of existing genomic material.

Novel traits that have evolved in a range of different taxa have recently been associated with taxonomically restricted genes (Khalturin et al., 2008; Johnson and Tsutsui, 2011; Ferreira et al., 2013; Looso et al., 2013; Harpur et al., 2014), and this includes the eusocial Hymenoptera (Simola et al., 2013; Wissler et al., 2013; Sumner, 2014). We predict that social parasites will harbor a higher proportion of new genes, gene functions, or novel gene networks relative to their free-living eusocial hosts. Additionally, ancestral genes may be modified substantially in function through modulation of their expression patterns, regulatory roles or protein production (Figure 2, area a).

Analyses of gene expression dynamics in *Polistes* social parasite brains at the pre-usurpation (OW), usurping (U) and post-usurpation (SU) phases of their life cycle (see Box 2), revealed significant changes in the expression of Arrestin (*Art)* and Apolipophorin (*Apo)* (Figure 3D). These genes are significantly up-regulated during usurpation—a critical period in a social parasite's life which, if not executed correctly during a narrow temporal window, could result in zero fitness (Turillazzi et al., 1990; Cervo and Turillazzi, 1996). During this phase, a novel behavior is exhibited—restlessness—(Ortolani et al., 2008), which is not found in the host (or non-host). No such variation of *Art* and *Apo* expression was detected in the host queens suggesting that these expression patterns are specific to the parasite's novel behavior, potentially due to the acquisition of regulatory mechanisms that enhance gene expression variability. Unbiased genome-wide RNAseq analyses are required to determine whether putative novel genes are also involved in usurpation behaviors. New genes may be important drivers of phenotypic evolution (Chen et al., 2013). Studies on social parasites and their hosts will therefore help identify some such novel genes, and facilitate further exploration of the role of novel genes in phenotypic evolution. Such phenotype-led gene discovery is likely to be a rich, untapped resource.

### Hypothesis 4: resistance to social parasitism in non-hosts will be reflected at the molecular level

Comparison of social parasites, hosts and non-hosts has the potential to reveal the molecular processes associated with host response to parasitism (Figure 2, area b), for example in host worker rebellions to the presence of social parasites in *Protomognathus americanus* ants (Achenbach and Foitzik, 2009), and resistance to social parasitism as found in sympatric non-host sister species (Figure 2 area c). In *Polistes dominula*, workers respond to parasite queens as if they were the host (mother) queen (Cervo et al., 1990; Cervo, 2006) suggesting that the parasite manipulates host workers successfully. However, recent work suggests that after several weeks of parasitism, workers are able to detect and respond to the parasite as they show some level of ovarian development, perhaps priming themselves for direct reproduction (Cini et al., 2014). Examining the molecular changes that take place in workers over the social parasite's life cycle may reveal important insights into the dynamic interactions of host and social parasite genomes, in a similar way to pathogens and their hosts (Riddell et al., 2011; Dybdahl et al., 2014).

Non-host sister species that occur sympatrically to the host in parasitized populations are powerful models for studying the molecular basis of social parasite resistance. For example, the free-living leafcutter ant *Acromyrmex octospinosus* co-occurs with its sister species *Acromyrmex echinatior*, and yet is resistant to parasitism by *Acromyrmex insinuator* (Sumner et al., 2004a; Box 1A); *Polistes nimphus* occurs alongside *P. dominula* and is resistant to invasion by *P. sulcifer* (Cervo, 2006; Box 1B). Phenotypically, there is no explanation for why co-occuring close relatives of hosts and social parasite are not also vulnerable to social-parasitism. We hypothesize that there will be key differences in the transcriptional and/or regulatory processes of hosts and non-hosts, which may confer resistance to non-hosts (Figure 2, area c). These may include novel processes (or novel usage of conserved genes) that have evolved in the non-host since speciation. Functional genomics (e.g., RNAi, cross-species expression experiments) provide powerful tools to test candidate genes or regulatory elements involved resistance.

## Conclusions and future perspectives

Comparative genomic analyses of obligate social parasites with their eusocial hosts and non-hosts are powerful approaches to studying losses and gains in phenotypic evolution. These analyses promise important insights into how genomes give rise to phenotypic diversity. We outline two scenarios for the evolution of social parasites from their eusocial ancestors. The scant data available to date suggest that the social parasite phenotype is distinct from their eusocial ancestor counter-part (i.e., eusocial queens). Social parasites therefore may not evolve through simple “deletion” (silencing) of the worker phenotype and its associated molecular functions (West-Eberhard, 2005). Based on recent empirical findings on the molecular basis of phenotypic evolution in other organisms, we predict that the evolution of new genes as well as the re-use of old ones will be important in the generation of the novel traits that characterize this new phenotype. We also predict that the full social parasite phenotype (defined as a combined consideration of the behavioral and molecular phenotype, Nachtomy et al., 2007) will be more complex than perceived from classical behavioral studies. Crucially, social parasites may retain the machinery for detecting and responding to the environment, just like their social ancestor and their free-living social hosts. The molecular processes associated with response to the environment, rather than behavior, are likely to be conserved (e.g., toolkit genes).

Our model and predictions are preliminary, but are relevant more widely to non-hymenopteran social parasites, as social parasitism of parental care has evolved multiple times in different taxa of the animal kingdom, e.g., birds (Davies, 2000); lycaenid butterflies (Fiedler, 2006); freshwater fishes (Baba et al., 1990). In each case, the social parasite is a highly specialized species that has lost the traits associated with caring for its own young, and evolved new traits that enable it to successfully insinuate its young into the home of its chosen host. More generally, our framework may also be relevant to phenotypic evolution in non-social parasites that are closely related to their hosts, such as in fungi, red algae and mistletoe, cynipids wasps, gall inducing aphids (West-Eberhard, 2003) and parasitoids (e.g., *Nasonia*, Werren et al., 2010).

### Conflict of interest statement

The authors declare that the research was conducted in the absence of any commercial or financial relationships that could be construed as a potential conflict of interest.
