# Supplementary material for: Social parasitism and the molecular basis of phenotypic evolution
Source: Front Genet. 2015 Feb 18;6:32. doi: 10.3389/fgene.2015.00032 (PMC4332356; doi:10.3389/fgene.2015.00032)
Supplement: Supplementary file 1 [file DataSheet1.PDF]

SUPPLEMENTARY MATERIAL
Methods for supporting data in Figure 3

Samples collection
Wasp samples were collected from Central Italy in June 2005 and 2009. Unparasitized colonies were collected from the field and parasitized colonies were obtained either by field collection or laboratory usurpations. Overwintering hosts and social parasites were collected from their hibernation sites while they were still in diapause, in March to April 2009. Wasp phenotype was assessed through observation of **i**ndividual behaviours in the field and/or laboratory. Wasps were killed directly into RNAlater (AMBION) in situ and stored at -20°C until qPCR analyses.

RNA extraction and quantitative-PCR
Total RNAs were isolated with TRIZOL (Invitrogen) from individual dissected brains and DNase treated (Turbo, PROMEGA). Quantification was conducted using a Nanodrop and absence of RNA degradation was checked on agarose gel. First strand cDNAs were synthesized with 1µg of total RNA and reverse transcribed with Retrotranscriptase kit (AppliedBiosystem). cDNAs were amplified by quantitative PCR using the LightCycler- DNA Master SYBR Green I kit (AB) and the following primers:
- HSP-Heat shock protein (70kb) 5'-CTGTTCTTAGCGGCAATGGTC-3' and 5'-TGGACAAATCTACTGGCAAGGAG-3',
- 09-Insulin growth factor 5'-TGCCATACTTTTTCGATGGGT-3' and 5'-GCGTTCCACCGATAATAGCTG-3',
- 15-Tubulin alpha1 chain 5'- AGCACCATCGAATCGTAAGGA-3' and 5'-ACATTCCGACTGCGCATTTAT-3',
- 26-Apolipophorin 5'-AGCACGAATTTCGCCAATAAA-3' and 5'-CGCATCTGGAATTTGAGCATT-3',
- 28-MRJP 5'-GCTAGACCAACGACACCATCATC-3' and 5'-TTCGCGAAAAATCCAAAAGC-3',
- 32-Arrestin 5'-TCGCAGTGTTGCACCACAA-3' and 5'-TCACTTTCTCTCAGGGCAAACTT-3'.

Since primer sets are directed on gene regions where both species share >90% similarity, comparable amplification efficiencies were obtained in both species. Gene specific standard curves were established using serially diluted cDNAs from a mixture of both host and social parasite brain RNAs. Q-PCR was performed on a LightCycler STEP-ONE (AB). Three technical replicates were run for both samples and standards. CTs values were removed if amplification baseline was 15< or >35 and if melting curves pattern were not sigmoidal. Selected CTs were submitted to Q/C of Q-base software and normalization against tubulin.

Statistical analyses
Statistical analyses were performed using PRISM and SPSS. Quantitative outliers were removed using ROUT method from PRISM. PRISM uses a robust nonlinear regression analysis to remove the outliers through the false discovery rate approach and perform ordinary least-squares regression on the remaining data (Motulsky and Brown, 2006). All data were log transformed. Statistical tests used are reported as appropriate in Figure 3.
